# Initial intracranial pressure as a prognosticator in head-injured patients undergoing decompressive craniectomy

**DOI:** 10.18632/oncotarget.11632

**Published:** 2016-08-26

**Authors:** Hua Liu, Rong Xu, Jian Yang, Guanghui Ren, Shengxue He

**Affiliations:** ^1^ Department of Neurosurgery, The First People's Hospital of Kunshan Affiliated with Jiangsu University, Suzhou, Jiangsu, China; ^2^ Department of Pediatric Surgery, The First People's Hospital of Kunshan Affiliated with Jiangsu University, Suzhou, Jiangsu, China; ^3^ Department of Neurosurgery, The Affiliated Brain Hospital, Nanjing Medical University, Nanjing, Jiangsu, China

**Keywords:** initial intracranial pressure, traumatic brain injury, decompressive craniectomy

## Abstract

**Purpose:**

To examine the prognostic discrimination and prediction of initial intracranial pressure (ICP) in patients with traumatic brain injury (TBI) undergoing decompressive craniectomy (DC).

**Results:**

The relationship between the initial ICP value and prognosis was quantified, and higher values indicated worse patient outcomes. Univariate analysis showed that the initial ICP value was significantly associated with mortality (odds ratio: 1.272, 95% confidence interval: 1.116-1.449; P<0.001) and unfavorable outcomes (odds ratio: 1.256, 95% confidence interval: 1.160-1.360; P<0.001). After adjustment for related outcome predictors of TBI in multivariate regression, the initial ICP value remained an independent predictor of unfavorable outcomes (odds ratio: 1.251, 95% confidence interval: 1.140-1.374; P=0.015) and mortality (odds ratio: 1.162, 95% confidence interval: 1.093-1.321; P=0.019).

**Methods:**

A retrospective study was conducted in 133 TBI patients after DC. Initial ICP was defined as the first ICP recorded during surgery. Mortality and Glasgow Outcome Scale score at the end of follow-up were used as outcome measures. Unfavorable and favorable outcomes were classified by a Glasgow Outcome Scale score of 1 to 3 and 4 to 5, respectively. We used binary logistic and proportional odds regression for prognostic analyses.

**Conclusion:**

For TBI patients undergoing DC, the initial ICP value provides great prognostic discrimination and is an independent predictor of unfavorable outcomes and mortality. We suggest that the initial ICP be included as a prognosticator in the overall assessment of prognosis of head-injured patients after DC.

## INTRODUCTION

Traumatic brain injury (TBI) constitutes the major cause of death and severe disability among young people. Intracranial lesions with intractable intracranial hypertension and malignant brain swelling in severe TBI patients continue to have devastating effects. A number of studies showed that decompressive craniectomy (DC) is an effective means of controlling high intracranial pressure (ICP), especially in patients with intraparenchymal lesions [1, 2]. DC is being more frequently performed; hence, the prediction of postoperative outcomes is highly important in neurosurgical practice. Glasgow Coma Scale (GCS) score, the main tool for clinical assessment of TBI, is correlated with the outcomes after DC [3]. However, it is sometimes difficult to count the GCS score because head injured patients are frequently drunk, sedated, or intubated.

ICP monitoring remains the cornerstone of acute neurological treatment after TBI and has become standard procedures in most large trauma centers [4, 5]. Recently, several studies have shown that the presence and frequency of high ICP are predictive factors in the outcome of TBI [6, 7]. However, few data are available regarding predictive value of ICP in TBI patients after DC. Initial ICP, which is the first measured ICP value, can reflect the extent of primary and secondary insults. The ability to predict prognosis after TBI on the basis of initial ICP values would aid in defining patients who might benefit from more complicated monitoring. Therefore, in this study, we retrospectively collected clinical data and quantified the relationship between the initial ICP value and prognosis after DC for TBI.

## RESULTS

### Patient characteristics

The clinical features and results of all patients are presented in Table 1. The 133 TBI patients who underwent DC included 107 male and 26 female patients. The mean age was 47.16±16.03 years (range, 14-85 years). The mechanisms of head injury were as follows: 102 traffic accidents, 29 falls, and 2 other causes. At admission, the mean ISS was 21.72±12.51 (range, 9-66). Neurological assessment before DC showed 34 patients (25.6%) with a GCS score of 3 to 5, 71 patients (53.4%) with a GCS score of 6 to 8, and 28 patients (21.1%) with a GCS score of 9 to 15, the mean GCS was 7.11±2.05 (range, 3-12). Pupillary examination identified 40 patients with two reacting pupils, 74 patients with one reacting pupil, and 19 patients with both nonreacting pupils. According to the Rotterdam CT classification, the number and percentage of patients with scores of 2, 3, 4, 5, and 6 were 4 (3.0%), 11 (8.3%), 18 (13.5%), 58 (43.6%), and 42 (31.6%), respectively.

**Table 1:** Clinical characteristic of 133 head-injured patients undergoing decompressive craniectomy

Variable	
**Mean age, y, ± SD**	47.16 ± 16.03
**Sex, no. (%)**	
Male	107 (80.5)
Female	26 (19.5)
**Mechanism of head injury, no. (%)**	
Traffic accident	102 (76.7%)
Fall	29 (21.8%)
Other	2 (1.5%)
**Mean ISS at admission, ± SD**	21.72 ± 12.51
**GCS score before DC, no. (%)**	
3-5	34 (25.6)
6-8	71 (53.4)
9-15	28 (21.1)
**Pupil reactivity before DC, no. (%)**	
Both reacting	40 (30.1)
One reacting	74 (55.6)
Both nonreacting	19 (14.3)
**Rotterdam CT score, no. (%)**	
2	4 (3.0)
3	11 (8.3)
4	18 (13.5)
5	58 (43.6)
6	42 (31.6)
**GOS at the end of follow-up, no. (%)**	
Unfavorable outcome	76 (57.14)
Favorable outcome	57 (42.86)
**Mean Initial ICP, mmHg, ± SD**	37.77 ± 12.65
**Time interval from head injury to DC,h,no. (%)**	
≤24	113 (85)
>24	20 (15)
**Hospital stay, d, ± SD**	30.28 ± 18.23
**Mortality at the end of follow-up, no. (%)**	11 (8.3)

ISS, Injury Severity Score; GCS, Glasgow Coma Scale; DC, decompressive craniectomy; CT, computed tomography; GOS, Glasgow Outcome Scale; ICP, intracranial pressure.

The initial ICP values of all 133 patients ranged from 19–83 mmHg, with a mean and SD of 37.77±12.65 mmHg. Unfavorable outcome was noted in 76 of 133 patients at the time of the evaluation. The mean initial ICP values were 43.74±12.75 (23-83) mmHg in the unfavorable group, whereas the mean initial ICP values were 29.81±6.83 (19-55) mmHg in the favorable group. The difference between the two groups was statistically significant (P<0.001).

A total of 113 patients (85.0%) were treated with emergency surgery within 24 hours after head trauma, whereas 20 patients (15.0%) were treated with delayed surgery more than 24 hours after injury. Unilateral frontotemporoparietal hemicraniectomy was performed in 123 patients (92.5%). Six (4.5%) and 4 (3.0%) patients underwent bilateral hemicraniectomy and bifrontal craniectomy, respectively.

The mean hospital stay was 30.28±18.23 days. The overall mortality rate was 8.3% at the end of the follow-up period. Additionally, 22 patients (16.5%) remained in a vegetative state, and 43 patients (32.3%) showed severe deficits. Forty-two patients (31.6%) had moderate deficits, and 15 patients (11.3%) showed good recovery and social reintegration.

### Initial ICP value vs prognosis

Table 2 shows the relationship between initial ICP value and prognosis of patients with TBI after DC. Higher ICP values were accompanied by higher rates of poor prognosis, and the frequencies of mortality and unfavorable outcome were 66.7% and 91.7%, respectively, when the ICP value>50mmHg.

**Table 2:** Initial ICP value vs mortality and unfavorable outcome

Initial ICP value (mmHg)	No. of Patients	Mortality, No. (%)	Unfavorable Outcome, No.(%)
**≤20**	3	0(0)	0(0)
**21-30**	39	0(0)	3(7.7)
**31-40**	56	0(0)	41(73.2)
**41-50**	23	3(13.0)	21(91.3)
**>50**	12	8(66.7)	11(91.7)

ICP, intracranial pressure.

Univariate analysis revealed that the initial ICP value was significantly associated with mortality (odds ratio: 1.272, 95% confidence interval: 1.116-1.449; P<0.001) as well as an unfavorable outcome (odds ratio: 1.256, 95% confidence interval: 1.160-1.360; P<0.001) (Table 3). After adjustments were made for sex, age, ISS, GCS score, Rotterdam CT score and pupil reactivity, the predictive effect of the initial ICP value on an unfavorable outcome (odds ratio: 1.251, 95% confidence interval: 1.140-1.374; P=0.015) remained substantial (Table 4).

**Table 3:** Univariate analysis of initial ICP value related to mortality and unfavorable outcome of head-injured patients undergoing decompressive craniectomy

	Mortality		Unfavorable Outcome	
	Odds Ratio (95% CI)	P Value	Odds Ratio (95% CI)	P Value
**Initial ICP value**	1.272 (1.116-1.449)	<0.001	1.256 (1.160-1.360)	<0.001

ICP, intracranial pressure.

**Table 4:** Multivariate analysis of potential predictors related to mortality and unfavorable outcome of head-injured patients undergoing decompressive craniectomy

	Mortality		Unfavorable Outcome	
	Odds Ratio (95% CI)	P Value	Odds Ratio (95% CI)	P Value
**Sex**				
Female	1.000		1.000	
Male	0.753(0.195-2.678)	0.497	0.673(0.184-2.455)	0.548
**Age**	1.030(0.958-1.113)	0.784	1.006(0.969-1.044)	0.772
**ISS**	1.103(0.912-1.135)	0.413	1.021(0.975-1.068)	0.381
**GCS score before DC**	0.758(0.563-0.952)	0.016	0.517(0.318-0.843)	0.008
**Pupil reactivity**				
One reacting	1.000		1.000	
Both reacting	0.000(0.000-0.000)	0.999	0.221(0.013-3.735)	0.438
Both nonreacting	1.867(0.312-9.157)	0.368	1.979(0.353-11.086)	0.296
**Rotterdam CT score**	4.584(0.984-18.436)	0.537	2.324(1.096-4.927)	0.028
**Initial ICP**	1.162(1.093-1.321)	0.019	1.251(1.140-1.374)	0.015

ISS, Injury Severity Score; GCS, Glasgow Coma Scale; ICP, intracranial pressure.

## DISCUSSION

Decompressive craniectomy reduces medically refractory intracranial hypertension and is a valuable tool in the management of severe head injury [8, 9]. However, the prognosis of patients after DC is highly variable. The aim of this study was to determine the value of initial ICP measurement in predicting prognosis of head-injured patients undergoing DC. Based on this study, the percentage of mortality and unfavorable outcomes gradually increased with the increase in the initial ICP. The initial ICP value was significantly associated with mortality and unfavorable outcomes, and proven to be an independent predictor of unfavorable outcomes and mortality after adjusted with relevant clinical characteristics in moderate and severe TBI patients.

Several studies have found that the presence of high ICP is a predictive factor for severe head injury outcomes [7, 10–12]. However, the relationship between initial ICP and outcome is less clear. Furthermore, the effect of initial ICP in TBI patients after DC is never reported. This is the only study that has evaluated the predictive value of initial ICP for prognosis of patients undergoing DC. Initial ICP values tend to reflect the extent of primary and secondary brain injury. If the initial ICP value of the patients was high, although ischemic and hypoxic lesions cannot be observed through imaging, varying degrees of hypo-perfusion could occur and the brain microenvironment could also be damaged to varying extents. Additionally, even if ICP returns to a normal level after craniectomy, cytotoxic cerebral edema and vasogenic cerebral edema can occur later and result in unfavorable outcomes [13, 14]. In clinical practice, quantitative prognostic estimates are of particular importance to the heterogeneous condition of TBI and can be applied to clinical decision making and assessment of the need for long-term care. A recent study presented clinical severity of intracranial injuries (GCS score), CT abnormalities and systemic insults (hypoxia and hypotension) as relevant predictive factors for outcomes in patients with moderate and severe TBI. We obtained the similar result that GCS score, CT abnormalities and the initial ICP values are independent predictors of unfavorable outcomes. However, Yuan et al found that the predictive value of initial ICP for unfavourable outcome was poor [15]. This could be attributed to the differences in TBI patients enrolled. They excluded the patients had dilated and unreactive pupils and hematomas or edemas which required surgery in a short time, whereas we focused on patients who had DC to treat hematomas or edemas and malignant brain swelling despite maximal medical management.

Previous study in TBI showed that clinical severity had the highest prognostic value, followed by CT characteristics [7]. However, a significant number of head-injured patients are already intubated and sedated, especially those with severe injuries, and hence neurological evaluation becomes problematic. Verbal response is not possible because of the endotracheal tube, and sometimes facial or ocular injuries can interfere with pupil evaluation and eye opening. Limitation of motor response is difficult to interpret due to either neurological deterioration or the effects of myorelaxants. In addition, TBI is frequently associated with alcohol or drug intoxication. Therefore, a reliable assessment of the prognosis after TBI must be combined with objective information such as the CT scan findings or initial ICP values.

Although several clinical predictors of death and functional outcomes are recognized in the literature [7, 16–18], quantitative analysis of prognosis by using a relevant factor has not yet been performed. We described and quantified the relationship between initial ICP value and prognosis at the end of the follow-up period. The differences in the observed rates of unfavorable outcome between patients from the lowest and the highest value groups were up to 91.7% (range, 0.0%-91.7%). Therefore, our investigation confirmed that the initial ICP was useful and practical as a prognostic tool for the examination of patients undergoing DC for TBI.

## CONCLUSION

For head-injured patients undergoing DC that is usually combined with evacuation of traumatic mass lesions, the initial ICP provides great prognostic discrimination. Logistic regression analysis also shows that the initial ICP value is an independent predictor of unfavorable outcomes. Because the ICP monitoring system is relatively objective, necessary, and practical, we suggest that the initial ICP value be included as a prognosticator in the overall assessment of a TBI patient's clinical condition after DC.

## MATERIALS AND METHODS

### Data collection

From February 2013 to December 2015, 133 TBI patients underwent DC at Kunshan Hospital. The patients' charts were retrospectively reviewed after consent was obtained from our institutional review board. The data collected included demographic information, mechanisms of injury, Injury Severity Score (ISS), pupillary reactivity, and GCS score. CT findings were scored according to the Rotterdam CT classification as follows: (a) status of basal cisterns subdivided into normal (0), compressed (1), or absent (2); (b) midline shift subdivided into 0 to 5 mm (0) or more than 5 mm (1); (c) epidural hematoma subdivided into present (0) or absent (1); and (d) traumatic subarachnoid hemorrhage or/and intraventricular hemorrhage subdivided into absent (0) or present (1) [19]. To add plus 1 to the sum score made the grading numerically consistent with the grading of the motor score of the GCS that stratified TBI into 6 categories of varying severity.

### Indications and techniques

Operative records were reviewed to determine surgical indications and details. There were 2 clinical scenarios in which DC for TBI was performed. The first scenario involved patients who had DC as part of an operation to treat a hematoma or edema or resective surgery for diffuse injury. The second was that patients had DC for refractory malignant brain swelling despite maximal medical management. Intraventricular ICP monitoring catheters (Codman Microsensors ICP Transducer, Codman & Shurtleff, Raynham, MA) were placed in all patients in this study. Initial ICP was defined as the first recorded ICP before DC. When brain swelling was limited to one cerebral hemisphere, unilateral hemicraniectomy was performed. For bilateral hemispheres or frontal swelling, bilateral hemicraniectomy or bifrontal craniectomy was chosen, respectively. Hemicraniectomy limits included (a) superior, 2 cm of the lateral edge of the superior sagittal sinus; (b) inferior, at level of the middle cranial fossa floor at the origin of the zygomatic arch; (c) anterior, frontal to the midpupillary line; and (d) posterior, 3 cm posterior to the external acoustic meatus. Bifrontal craniectomy extended posteriorly to just approximately 2 cm in front of the coronal suture and laterally to the floor to the middle fossa. The dura mater was opened, and the opening was extended to the bone margins in a stellate or semicircular fashion. The brain surface was covered loosely by the remaining dura or artificial dural substitutes without a watertight closure [20].

### Outcome assessment

Neurological outcome was determined according to the Glasgow Outcome Score (GOS) as follows: 1 = dead; 2 = vegetative state with inability to interact with the environment; 3 = severe disability with inability to live independently but the ability to follow commands; 4 = moderate disability with the ability to live independently but inability to return to work or school; and 5 = good recovery with the ability to return to work or school [21]. Prognostic evaluations were determined using the GOS assessment 6 months after the trauma. GOS evaluations were performed by physicians either in person or via telephone. A GOS of 1–3 was categorized as an unfavorable outcome, while a score of 4–5 was deemed a favorable outcome.

### Statistical analysis

Data were analyzed using SPSS version 19.0 (SPSS Inc, Chicago, Illinois). The strength of the association between the initial ICP value and prognosis after DC for TBI was examined by a univariate analysis using binary logistic regression models. Two separate dichotomies were considered: mortality vs survival and unfavorable vs favorable outcome. Results are expressed as odds ratios with 95% confidence intervals. Multivariable proportional odds regression was performed to adjust for established predictors of outcome after TBI (sex, age, ISS, GCS score, Rotterdam CT score, initial ICP value, pupil reactivity).
